# Preparation of Thermoplastic Polyurethane/Multi-Walled Carbon Nanotubes Composite Foam with High Resilience Performance via Fused Filament Fabrication and CO_2_ Foaming Technique

**DOI:** 10.3390/polym15061535

**Published:** 2023-03-20

**Authors:** Huijing Guo, Naveen Thirunavukkarasu, Suhail Mubarak, Huang Lin, Chen Zhang, Yonggui Li, Lixin Wu

**Affiliations:** 1 School of Chemistry, Fuzhou University, Fuzhou 350116, China; 2 CAS Key Laboratory of Design and Assembly of Functional Nanostructures, Fujian Key Laboratory of Nanomaterials, Fujian Institute of Research on the Structure of Matter, Chinese Academy of Sciences, Fuzhou 350002, China; 3 Fujian Key Laboratory of Novel Functional Textile Fibers and Materials, Minjiang University, Fuzhou 350108, China; 4 Department of Chemical and Biomolecular Engineering, Chonnam National University, Yeosu 59626, Republic of Korea; 5 School of Materials and Chemistry Engineering, Minjiang University, Xiyuangong Road No. 200, Fuzhou 350108, China; 6 Industrial Design Institute, Minjiang University, Xiyuangong Road No. 200, Fuzhou 350108, China

**Keywords:** fused filament fabrication, thermoplastic polyurethane, multi-walled carbon nanotubes, CO_2_ foaming, mechanical properties, sensor

## Abstract

Wearable flexible sensors with high sensitivity and wide detection range are applied in motion detection, medical diagnostic result and other fields, but poor resilience and hysteresis remain a challenge. In this study, a high-resilience foam sensor was prepared through a combination of additive manufacturing and green physical foaming method. The conductive filaments were prepared by using MWCNTs-modified TPU by the physical method of melt blending. Samples were prefabricated using the FFF printer and then saturated with CO_2_ in an autoclave before being removed and heated to foam. The composite foam effectively reduced residual strain, demonstrating the high resilience of the 3D-printed composite materials with a foam porous structure. The residual strain of the sample before foaming was >6% after a single cycle, and then gradually increased. The residual strain of the foamed samples is less than 5%. In addition, composite foam has high sensitivity and can monitor subtle pressure changes (0~40 kPa). The sensing performance of the composite foam was evaluated, and the current signal remained stable under different loading rates and small compression strains (2~5%). By using this highly resilient conductive composite material, a hierarchical shoe insole was designed that successfully detected human walking and running movements.

## 1. Introduction

Additive manufacturing or 3D printing is a technology based on the digital production of defined structures and geometric figures. It has been rapidly developed in several fields in recent years due to its advantages of controllable operation and high degree of design freedom. Compared with traditional molding methods, the selection of materials in the 3D printing process has more flexibility and functionality. For example, flexible electronic devices with customized structures can be designed through 3D printing by selecting flexible substrates with conductive materials. 3D printing can manufacture functional materials with complex macroscopic and controllable microscopic structures, which can greatly enhance the advantages of future biomedical sensing devices, including lower detection limits, higher sensitivity, larger sensing range and real-time diagnosis. Many studies have shown that flexible electronic devices show great potential applications in the fields of electronic skin, biomedicine and wearable electronic devices due to their high stretchability, high resilience and lightness [1,2,3,4]. As the core component of wearable electronic devices, flexible sensors can convert external mechanical changes, temperature, humidity and other stimulus deformation into electrical signals or other signals according to certain rules during the reconstruction process based on conductive network [5]. At the same time, wearable electronic sensors are currently considered as the next generation of tools for various healthcare applications. The current preparation methods of flexible sensors include atomic deposition, lithography, spraying and sputtering, etc. [6]. Due to the long-time program control, the large number of materials used and 2D plane structure limitations, they cannot meet the growing demand of human for customizable flexible wearable electronic devices. 3D printing flexible sensors can not only reduce the development and use of raw materials, but also can be customized according to the requirements of the construction of flexible sensors with high sensitivity and low hysteresis [7,8]. 3D printing flexible sensors can be divided into fused filament fabrication (FFF) [9,10], digital light processing (DLP) [11] and direct ink writing (DIW) [12]. FFF is a process in which filament of thermoplastic polymer is heated and melted at nozzle and then built up layer by layer. It is widely used due to its advantages of simple operation, fast printing and being able to process many materials. FFF means that a variety of thermoplastic polymers can be used for heat-induced melting and extrusion; polymers commonly used in FFF processing include Thermoplastic Polyurethane (TPU), Poly(ether-ether-ketone) (PEEK), Acrylonitrile Butadiene Styrene (ABS), Poly(lactic acid) (PLA) and Polycarbonate (PC) etc. Due to its excellent flexibility and ductility, as well as wear resistance and tear resistance, TPU are widely used in the modification of flexible sensors through conductive fillers. Christ et al. [13] and Hohimer et al. [14] mixed TPU with MWCNTs using twin screws to prepare printable wires, which were stacked and molded by an FFF printer to prepare resistive flexible sensors with good electrical conductivity and stretchability. Chen et al. [1] used biaxially stretched MWCNTs/TPU nanocomposites to prepare strain sensors with high performance. The results show that the biaxial stretching process enhances the uniform dispersion and plane orientation of the MWCNTs, which improves the crystallinity of the material and the sensing property of the sensor. Josef F Christ et al. [10] used multi-material fused filament fabrication tandem 3D printing to generate uniaxial and biaxial sensors with different conductive pattern designs. The sensors were subjected to a series of cyclic strain loads. Results show excellent piezoresistive response and cyclic repeatability in the axial and lateral directions and for strains of up to 50%.

The traditional foaming method is to add a chemical blowing agent (CBA) such as azodicarbonamide (AC) into the polymer and heat it to release gas during decomposition, which guides cell nucleation and growth. The size of the CBA and its dispersion in the matrix can hinder the reduction of cell size, and affect the foaming performance of the polymer. In addition, CBA can generate harmful substances during foaming and remain inside the foam product, so this is a trend towards using green foaming methods. Using physical blowing agents (PBA) for foaming (for example N_2_, CO_2_) is not only friendly to the environment and leaves no chemical residue, but also has low cost and high efficiency. Physical foaming technology has achieved great success in preparing high-performance thermoplastic foam with microporous or nanoscale porous structure, reducing cell size to below 100 µm, thereby improving the mechanical properties of thermoplastic materials. Currently, there are three foaming methods: batch foaming; injection foaming; and extrusion foaming. Elastomers have hard segment (HS), low crystallinity, low modulus, excellent elasticity and a high gas diffusion rate. They exhibit unique cell nucleation, growth and cell structure stabilization during physical foaming, and exhibit post-foaming shrinkage behavior [15,16]. Carbon-based nanomaterials are typically combined with various polymers, and need to consider the size and structure of the nanomaterials to ensure their even dispersion in the matrix for obtaining flexible and stable strain sensors [17]. The commonly used carbon materials include carbon nanotubes, graphene, carbon black and their mixed materials [18]. Carbon foam has a unique 3D porous structure, including both an open-cell and closed-cell structure, with excellent properties such as high electrical conductivity, high thermal conductivity, low density, high mechanical strength, high adsorption capacity and an electromagnetic shielding effect [19]. Gunasekaran, H.B. et al. deposited graphene on the surface of the sample by in-situ spraying during the fusion deposition molding process, and then physically foamed it. However, such deposited nanoparticles on the surface may form off on the surface over time, or fall off due to expansion during foaming [20]. 

Therefore, this work choses to fuse blended nanoparticles in the substrate to make them more compatible with the substrate. MWCNTs have a nanoscale hollow tubular structure obtained by curling single or multiple layers of graphene nanosheets, and due to their excellent aspect ratio, they are an ideal material for preparing flexible strain sensors. Additionally, there is anisotropy in the vertical direction during the FFF printing process. In this work, a green foaming method is proposed to prepare the high resilience foam sensor. Samples were prefabricated using the FFF printer and then saturated in the autoclave with CO_2_. The gas saturation condition was 5 MPa/25 °C and the foaming temperature was 80 °C. The dispersion of MWCNTs in the composite and the relationship between the content of MWCNTs and the foaming behavior of the composite were studied. The mechanical and sensing properties of the composite foam were evaluated. In addition, foam sensor as an insole can monitor the change of motion amplitude well, which is expected to become the future development trend of the combination of FFF technology and CO_2_ foam technology to produce customized and lightweight wearable sensors.

## 2. Materials and Methods

### 2.1. Materials

Polyester-based TPU-65A with a density of 1.18 g/cm^3^ was obtained from Wanhua Chemical Group Co., Ltd., Yantai, China. HQNANO-CNTs-010-0 multi-walled carbon nanotubes with a density of 2.1 g/cm^3^ (Purity > 95%, Length: 3–12 μm, OD: 8–15 nm) were obtained from Suzhou Tanfeng Graphene Technology Co., Ltd., Suzhou, China. High-purity CO_2_ gas (Purity > 99%) was obtained from Fuzhou Xinhang Industrial Gas Co., Fuzhou, China.

### 2.2. Filament Preparation and 3D Printing

To prepare high-quality FFF parts, the quality of the filament and the parameter settings during the printing process are key components of the process. Firstly, TPU masterbatches and MWCNTs are dried at 80 °C for 2 h, using the HAAKE-PolyLab twin-screw extruder to melt the co-blended masterbatch, and then the filament is extruded, collected by a downstream winder and pelletized [21]. The process was cycled twice to enable the MWCNTs to be uniformly dispersed in the TPU matrix. The content of the prepared filament MWCNTs is 0, 1, 2, 4 wt%, and controlled filament diameter of 2.25 ± 0.05 mm. Simplify software was used to design sample models and set printing parameters. The flexible F350 3D printer was used to print samples. By adjusting the appropriate printing parameters, the printing samples with good interlayer bonding were obtained. The samples used for tensile test were 70 mm (length) × 13 mm (width) × 1.8 mm (thickness) with a 70% filling rate, and the honeycomb samples used for compression test were 25 mm (length) × 25 mm (width) × 15 mm (thickness) with a 30% filling rate (printed and foamed samples are attached in Supplementary Figure S4.).

### 2.3. CO_2_ Batch Foaming Process

According to the specific preparation methods reported in previous literature [16], the printed samples were first placed into an autoclave connected with CO_2_ gas, and the samples were saturated in CO_2_ gas for 30 min at a saturation pressure of 5 MPa. After finishing the saturation process, the samples were quickly transferred to the hot water bath at 80 °C for 30 s of the expansion process. Cell growth process is presented in Supplementary Figure S1. This was then removed, and the foam was placed in room-temperature water for 30 s to stable. Finally, the foam was removed and placed in an oven to dry the moisture. The samples were analyzed starting 24 h after preparation to ensure the stability of the foam samples, and then characterized. The TPU/MWCNTs conductive foam with different MWCNTs content was named as TPU/xMWCNTs. To prepare TPU foam and TPU/MWCNTs-conductive foam with a uniform contour size, specific foaming conditions of 80 °C/30 s are required.

### 2.4. Testing and Characterization

The dispersion of MWCNTs in the prepared filaments and the cell morphology of the 3D-printed samples after foaming were observed using a scanning electron microscope (SU8010, Hitachi, Tokyo, Japan) and a benchtop scanning electron microscope (COXEM-EM-30Plus, China), respectively. Thermogravimetric analysis was carried out on the instrument STA449F3 comprehensive thermal analyzer under a nitrogen atmosphere, the heating rate was 10 °C/min and the test temperature range was 30–800 °C. Analysis of TG is presented in Supplementary Figure S3. The tensile and compressive properties with a load of 1 kN were evaluated using a universal testing machine (AGX-100 plus, Shimadzu, Kyoto, Japan), with a tensile loading rate of 50 mm/min. The tensile stress of the composite foam was obtained by dividing the tensile load by the cross-sectional area of the sample. The rate of compression loading is 30 mm/min, and the compressive stress of the composite foam was obtained by dividing the compressive load by the cross-sectional area of the sample. Since the foam sample undergoes porous deformation during stretching and compression, it is difficult to calculate the exact cross-sectional area, so the nominal stress is used in this test, and its length is measured before the test to calculate the cross-sectional area. A universal testing machine and a digital multimeter (RIGOL, DM3068, Guangzhou, China) were used to measure the signal changes of TPU/MWCNTs during compression to characterize the sensing performance of the porous flexible foam sensor [22].

## 3. Results

### 3.1. Microscopic Morphology

**Figure 1:**
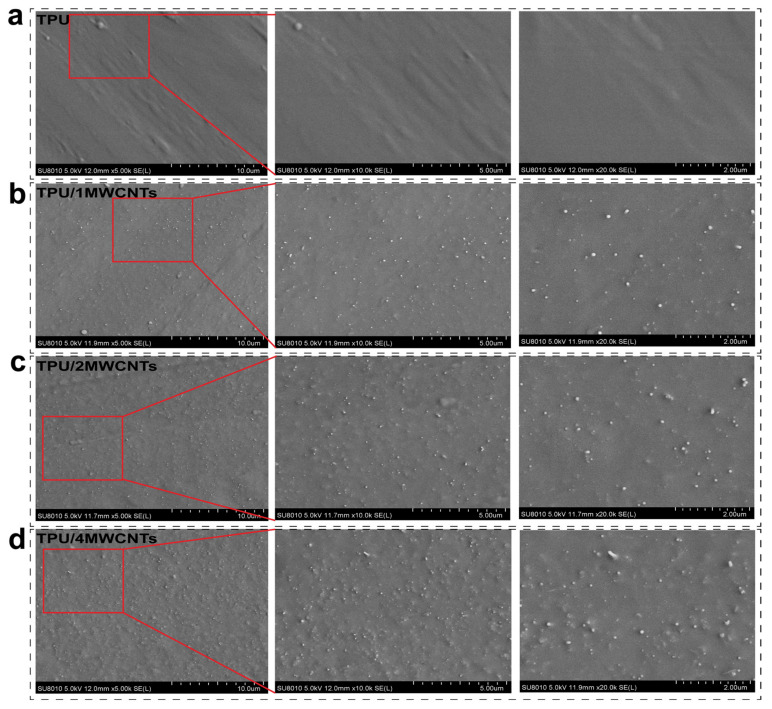
(**a**–**d**) Microscopic morphology of TPU and TPU/MWCNTs filaments with different MWCNTs content (0, 1, 2, 4 wt%). (Magnification: 5k, 10k, 20k).

Figure 1 shows the dispersion of MWCNTs in the TPU matrix. During the experiment, MWCNTs and TPU were melt-blended and extruded three times in a twin-screw extruder. It can be seen that MWCNTs are uniformly dispersed in TPU in the matrix without obvious agglomeration, and have good interfacial compatibility with TPU. MWCNTs have an excellent L/D ratio, so adding them as conductive filler in the matrix can provide more heterogeneous nucleation sites during the foaming process [23]. The FFF technique changes the porosity of the sample by changing the filling ratio of the printed sample. The printed samples are saturated in an autoclave connected with CO_2_ gas to form a polymer/gas homogeneous system. After the saturation, the cells are taken out by heating to induce cell nucleation and growth, and the sample showed obvious expansion. Figure S2 shows the SEM after foaming of the pure TPU injection sample and the print sample with 90% filling density. From the figure, it can be seen that the cells are small and not fully foaming, so the print model and filling density were adjusted and a honeycomb three-dimensional model (30% filling density) was chosen to allow the gas to enter inside the sample from every spatial dimension, while the filling density was reduced to provide a wider pathway [24]. The microcellular foaming process expanded the volume, and thus increased the size of the TPU honeycomb size. The formation of a microcellular structure within the selected range of foaming conditions is caused by gas diffusion-driven cell growth and cell shrinkage induced by gas escape. Elastomers have a high gas diffusion rate, and a large amount of gas escape can lead to volume shrinkage during cell growth, but there is no significant cell agglomeration in the TPU foam due to the TPU crystalline HS enhancing heterogeneous nucleation and reducing the shrinkage of foam samples [25].

**Figure 2:**
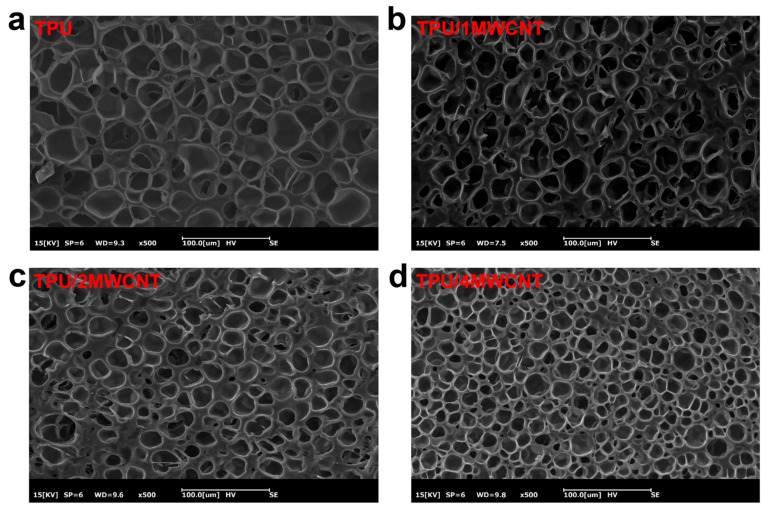
(**a**) Microscopic cells morphology of TPU foam; and (**b**–**d**) microscopic cells morphology of TPU/MWCNTs composite foam with different MWCNTs content (1, 2, 4 wt%). (Magnification: 500).

Figure 2a shows the cell morphology of pure TPU foam with a uniform size. Figure 2b–d shows the cell morphology of the composite conductive foam with added MWCNTs. It can be seen from Figure 2 that as the MWCNT content increases, the number of cells gradually increases and the diameter of cells gradually decreases. This is because the increasing MWCNTs content provides more heterogeneous nucleation sites during the foaming process. Therefore, adding MWCNTs as a conductive filler in the TPU matrix not only provides more heterogeneous nucleation sites during the foaming process, but also enhances the interface stability between MWCNTs and TPU by forming a pore structure.

### 3.2. Tensile Properties

**Figure 3:**
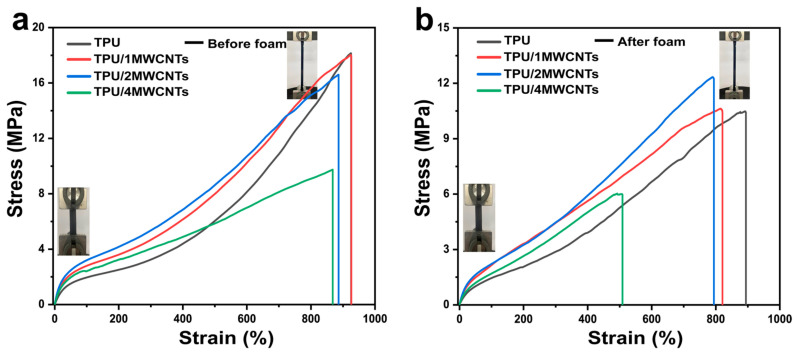
(**a**,**b**) Tensile stress-strain curves of TPU and TPU/MWCNTs composites with different MWCNTs content (0, 1, 2, 4 wt%) before and after foaming (Loading speed: 50 mm/min).

The mechanical properties of TPU and TPU/MWCNTs composite materials before and after foaming were evaluated by a tensile test. The printing density of tensile samples was 90%. The tensile stress-strain curve before foaming is shown in Figure 3a. When the MWCNTs content is 1 wt% and 2 wt%, the tensile stress and elongation at the break of the nanocomposites are slightly decreased, but still maintain high tensile strength and elongation at break [26]. As the MWCNTs content continued to increase, the tensile strength of TPU/4MWCNTs decreased significantly. This is because the large amount of MWCNTs added up to increase the internal agglomeration defects of the material, resulting in a decrease in the toughness of the nanocomposites, and the elongation at break could not exceed that of the pure substrate [27]. The tensile stress-strain curve before foaming is shown in Figure 3b; both the tensile strength and elongation at break decreased compared with that before foaming. However, the foam samples with low MWCNTs content still had higher tensile strength and elongation at break, and the tensile strength and elongation at break of TPU/4MWCNTs foam samples significantly decreased. The decrease in tensile strength and fracture elongation at break after foaming is due to the introduction of the microporous structure inside the sample, making it easier to deform under stress.

### 3.3. Compression Properties

The TPU honeycomb model has light weight, high anisotropy and unprecedented mechanical properties, such as high energy absorption capacity, a high stiffness to density ratio, vibration characteristics and elastic wave propagation controllability [28,29]. TPU foam shows good dynamic elasticity, high transient elasticity and a strong elastic recovery ability [30]. When introducing a cellular structure into the honeycomb model through foaming, the resilience characteristics of TPU foam components will be improved further. The honeycomb model is usually used for compression cycle testing. In this work, the compression properties of TPU and TPU/MWCNTs composite foam were measured and compared. Three characteristics can be observed from these curves: (1) all stress-strain curves are smooth and do not show fluctuations, indicating that the honeycomb model did not collapse or undergo plastic deformation during the test; (2) pure TPU foam and TPU/MWCNTs composite foam show three different linear deformation, platform and densification regions; and (3) the densification region shows a decrease in strain, an increase in platform stress and a decrease in platform length [25]. During cyclic compression, as the compressive strain increases, the area of hysteresis loop gradually increases and a significant hysteresis loop is produced between the loading and unloading curves of the cycle. This is caused by strain-softening behavior. This strain-softening behavior, known as the Mullins effect [31], has been widely reported in solid TPU, TPU foam and TPU honeycomb models. Typical stress-strain curves have three regions when subjected to strain, namely the initial elastic region, platform region and densification region. Viscoelastic materials exhibit a behavior called damping, which is obtained by the difference between the loading and unloading curves of the sample [32].

**Figure 4:**
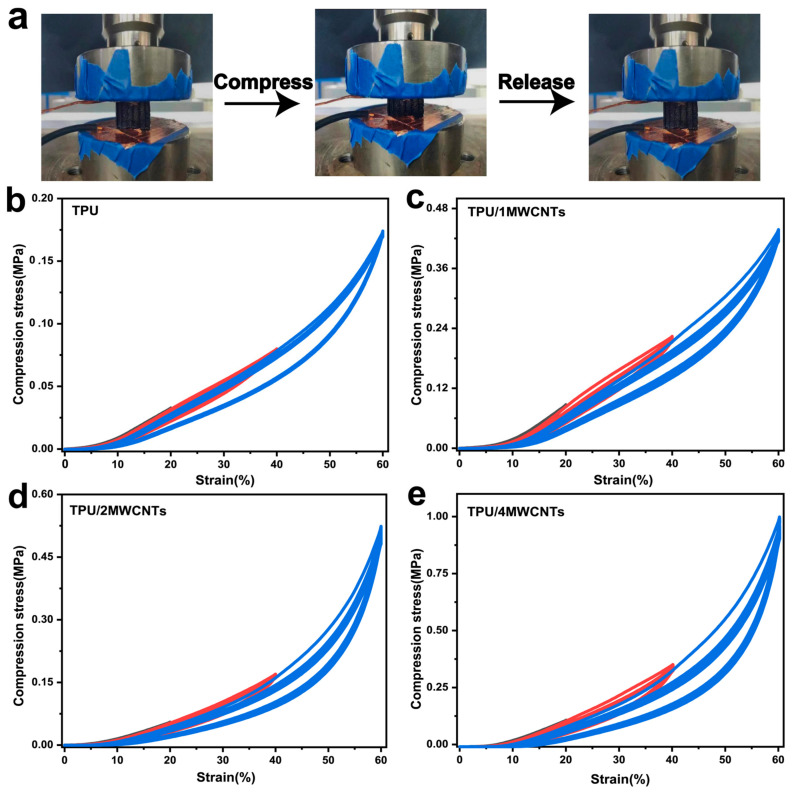
(**a**) The loading and releasing stages of the compression process; and (**b**–**e**) dynamic cyclic compression stress-strain curves of TPU foam and TPU/MWCNTs composite conductive foam with different MWCNTs contents (0, 1, 2, 4 wt%).

**Figure 5:**
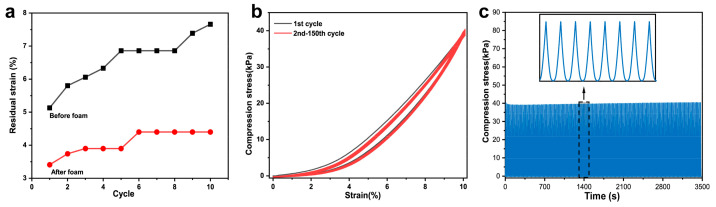
(**a**) A comparison of residual stress before and after foaming of TPU/4MWCNTs at 40% compression strain; (**b**) stability of TPU/4MWCNTs composite foam at 150 cycles of 40% compression; and (**c**) maximum stress maintained in TPU/4MWCNTs composite foam at 150 cycles of 10% strain compression.

Figure 4 shows the compressive stress-strain curves of TPU foam and TPU/MWCNTs composite conductive foam [20]. The cyclic loading and unloading process is repeated 10 times at 30 mm/min constant compression rate under strains of 20%, 40% and 60%. As can be seen from the compression curve of the TPU foam sample in Figure 4b, the small deformation area (<10%) shows an obvious linear change area. With the increase of compressive strain (10–35%), the growth of compressive stress gradually shows a gentle region. When gradually increasing the compressive strain range (35–50%), the curve reaches the dense zone and the compressive stress of the sample increases significantly [5]. At a strain of 60%, the required compressive stress is 0.17 MPa. With the increase of MWCNTs content (Figure 4c–e), the compressive stress gradually increases, and the maximum compressive stress of TPU composite foam with different MWCNTs content are 0.43 MPa, 0.52 MPa and 0.99 MPa [33]. The improvement in the mechanical properties of TPU/4MWCNTs composite foam can be attributed to the addition of more inorganic nanoparticles, which increase the overall rigidity and elastic modulus of the material. Among them, the compression strength of TPU/4MWCNTs at a large strain of 60% is only six times higher than that of pure TPU foam at the same strain. With the increase of MWCNTs content, the foam strength increases continuously and the foam cannot fully recover to the initial state after loading and releasing, but the residual strains were all in a low range. The TPU/4MWCNTs composites were compressed for 10 cycles before and after foaming at 40% strain. Figure 5a shows that the residual strain of the sample before foaming is >6% after a single cycle, and then gradually increases. The residual strain of the foamed samples is less than 5%. Figure 5b shows the stress-strain curves of TPU/4MWCNTs at 10% strain for 150 cycles of compression and the compression curves of the foam almost completely overlapped, and the maximum compressive stress remains at 40 kPa after the completion of the 150th cycle of compression test, indicating that the foam has a good resilience performance, as shown in Figure 5c.

### 3.4. Sensor Properties 

**Figure 6:**
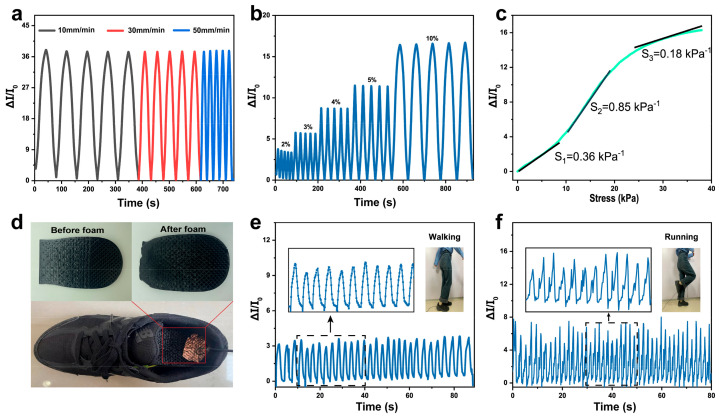
(**a**) Variation of relative current of TPU/4MWCNTs composite conductive foam at different compression rates; (**b**) variation of relative current of TPU/4MWCNTs composite conductive foam at small compression strains (2–10%); (**c**) pressure sensitivity of conductive composite foam; (**d**) TPU/4MWCNTs composite conductive foam as a plantar wearable sensor for gait recognition; and resistance responses for different gait patterns: (**e**) walking and (**f**) running.

Dynamic cyclic compression experiments were used to observe the changes of the current signals of TPU/4MWCNTs composite conductive foam, and upon further study of the sensing properties of TPU/4MWCNTs composite conductive foam, the relative current change is defined as ΔI/I_0_ = (I − I_0_)/I_0_. Figure 6a shows that TPU/4MWCNTs composite conductive foam exhibits stable and consistent signal peak changes under 10% strain applied at different compression rates. Figure 6b shows that when TPU/4MWCNTs composite conductive foam was compressed at the same rate (30 mm/min) but with different compression strains, ΔI/I_0_ increases from 3.37 to 16.60 in the compression strain range of 2–10%. Under small compression strains of 2–5%, ΔI/I_0_ can respond with stability, and the signal remains stable even with small strain changes, indicating that TPU/4MWCNTs composite conductive foam can not only detect small deformations, but also provide stable and reliable signal responses. Sensitivity is an important parameter to evaluate the sensing properties, and is defined as the relative change in the electrical signal divided by the slope of the applied pressure curve. Figure 6c shows the sensitivity change of TPU/4MWCNTs composite conductive foam at a small strain of 10%, as per the sensitivity calculation equation

S = d(ΔI/I_0_)/dP (1)

and with the applied pressure S increases, the trend increases and then decreases. As the pressure increases, the compression deformation of the foam gradually increases, continuously forming new conductive pathways. The strong interfacial interactions of the foam contribute to the enhancement of its sensitivity [34]. S increases to 0.36 kPa^−1^ under 0~10 kPa higher than that reported in previous literature of PDMS foam compressive stress and sensitivity variation of low-stress states in shear and normal directions of three-dimensional interfacial graphene foam [35,36]. As the pressure further increases, S decreases from 0.85 kPa^−1^ to 0.18 kPa^−1^; this shows that composite foam has high sensitivity and can monitor subtle pressure changes (0~40 kPa).

We further demonstrated the application of conductive foam as a wearable sensor in human motion monitoring. For example, porous foam elastic insoles that conform to the shape of the human foot were prepared using 3D printing and CO_2_ foaming techniques. The insoles are embedded into the soles of sports shoes and connected to two wires as pressure sensors, enabling the monitoring of motion amplitude and frequency. The foam samples used in the experiment are non-toxic, and are fixed using insulating tape during the test to avoid direct contact with the human body. A digital multimeter was connected to record the current response ΔI/I_0_ of the porous foam pressure sensor in real time, and the change in human gait is analyzed by the changing ΔI/I_0_. Figure 6d shows a sports shoe equipped with a layered porous foam insole as a sensor. Figure 6e,f shows the change curves of the current signal when the volunteer walks and runs; the ΔI/I_0_ when walking is about 3, which is smaller than the ΔI/I_0_ when running (5~8). The ΔI/I_0_ signal variation curve while walking is relatively stable, while the signal variation curve during running is larger in magnitude because the whole body is in vigorous motion. In addition, the ΔI/I_0_ signal change time was shorter for walking than for running, indicating that the magnitude of motion could be distinguished by the frequency of ΔI/I_0_ signal change. These results confirm that customized porous foam insoles prepared by 3D printing and foam technology can not only monitor the change of human motion amplitude, but also improve the comfort of human wear [37,38].

## 4. Conclusions

In summary, this work proposes a green method for preparing foam sensors with high resilience by combining fused filament fabrication and physical foaming techniques. The conductive filaments were prepared by using MWCNTs-modified TPU by the physical method of melt blending, which had good dispersion and interface compatibility. The foam prepared by high-pressure fluid technology has uniform pores, and can provide stable changes during deformation. The composite foam effectively reduces residual strain. The residual strain of TPU/MWCNTs composite material before foaming is greater than 6% after a single cycle, and gradually increases. However, the residual strain of the foaming samples is less than 5%. In addition, the composite foam has high sensitivity and can detect subtle pressure changes (0~40 kPa). The stability of the composite foam was evaluated, and it maintains a stable maximum stress of 40 kPa after 150 cycles at 10% strain. The sensing performance of the composite foam was evaluated, and a hierarchical shoe insole was designed using this high-resilience conductive composite material, which successfully detected human walking and running movements. By combining green physical foaming techniques, layered porous 3D-printed conductive materials could potentially be used to produce lightweight and customized wearable sensors. The batch foaming process in this work requires a good control of the time when the sample is taken out of the autoclave and put into the hot water bath after saturation, and there must be no human error. Therefore, in the future, a path can be designed at the nozzle to connect CO_2_ gas and foam at the same time when the material is extruded, in order to realize a one-step method to prepare thermoplastic foam with high design freedom.

## Data Availability

The authors confirm that the data supporting the findings of this study are available within the article.

## Supplementary Materials

The following supporting information can be downloaded at: https://www.mdpi.com/article/10.3390/polym15061535/s1, Figure S1, cell growth process; Figure S2(a–c), SEM after foaming of pure TPU injection sample; and (d–f) SEM after foaming of printed sample with 90% filling density; Figure S3, TG of TPU composites with different MWCNTs content; and Figure S4, printed and foamed samples.

## Abbreviations

FFF	Fused filament fabrication	ABS	Acrylonitrile Butadiene Styrene
DLP	Digital light processing	PLA	Poly(lactic acid)
DIW	Direct ink writing	PC	Polycarbonate
TPU	Thermoplastic Polyurethane	PEEK	Poly(ether-ether-ketone)
MWCNTs	Multi-walled carbon nanotubes	AC	Azodicarbonamide
PBA	Physical blowing agent	CBA	Chemical blowing agent
HS	Hard segment
