# Supplementary material for: Preparation of Thermoplastic Polyurethane/Multi-Walled Carbon Nanotubes Composite Foam with High Resilience Performance via Fused Filament Fabrication and CO2 Foaming Technique
Source: Polymers (Basel). 2023 Mar 20;15(6):1535. doi: 10.3390/polym15061535 (PMC10054835; doi:10.3390/polym15061535)
Supplement: Supplementary file 1 [file polymers-15-01535-s001.zip › polymers-2219028-supplementary.pdf]

Preparation of thermoplastic polyurethane / multi-walled car-bon nanotubes composite foam with high resilience performance via fused filament fabrication and CO_2 foaming technique

Huijing Guo [1,2,3], Naveen Thirunavukkarasu [2], Suhail Mubarak [4], Huang Lin [2], Chen Zhang [5,6,*], Yonggui Li [3,*], Jianlei Wang [2,3,7] and Lixin Wu [2,*]

1 School of Chemistry, Fuzhou University, Fuzhou 350116, China

2 CAS Key Laboratory of Design and Assembly of Functional Nanostructures, Fujian Key Laboratory of Nanomaterials, Fujian Institute of Research on the Structure of Matter, Chinese Academy of Sciences, Fuzhou 350002, China

3 Fujian Key Laboratory of Novel Functional Textile Fibers and Materials, Minjiang University, Fuzhou 350108, China

4 Department of Chemical and Biomolecular Engineering, Chonnam National University, Yeosu, Jeonnam 59626, Republic of Korea

5 School of Materials and Chemistry Engineering Minjiang University, Xiyuangong Road No.200, Fuzhou 350108.China

6 Industrial Design Institute, Minjiang University, Xiyuangong Road No.200, Fuzhou 350108, China

7 Engineering Research Center of Polymer Green Recycling of Ministry of Education, Fujian Normal University, Fuzhou 350007, China

* Correspondence: zhangchen@mju.edu.cn (C.Z.); ygwxjd@sina.com (Y.L.); xwu@fjirsm.ac.cn (L.W.)

Supporting Information

S1. Cell growth process

The intermittent CO_2 foaming process can be divided into four stages: 1) gas is injected into the polymer matrix under certain conditions to reach saturation state, forming a polymer/gas homogeneous system; 2) the sudden rise of temperature or sudden fall of pressure causes the gas in the homogeneous system reach a state of saturation, that is, the thermodynamic instability, which leads to cells nucleation; 3) the gas rapidly diffuses into the cells, and the cells grow; 4) cooling and stabilizing the cells structure.

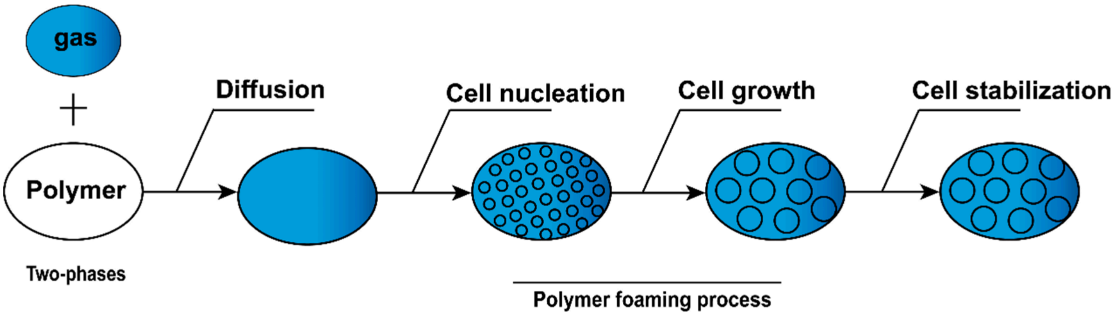

Figure S1. Cell growth process.

S2. Cell morphology of injection and printed sample

As can be seen from Figure S2a, there are only cells in the outer layer and no cells in the inner part (Figure S2b). This is because there is no pore path in the injection molding sample, so it is difficult for CO_2 gas to enter during the saturation of the autoclave. In addition, the gas entering the outer layer is difficult to escape due to resistance during the release process, and it cannot be fully foaming during the heating process.

The printed sample with a filling density of 90% has relatively uniform cells (Figure S2e). The pores in the figure are formed during the printing process and provide paths for gas to enter the sample. However, the bubble holes are not fully foamed (Figure S2f). For full foaming, the filling density can be reduced to provide more routes for the gas to enter the sample.

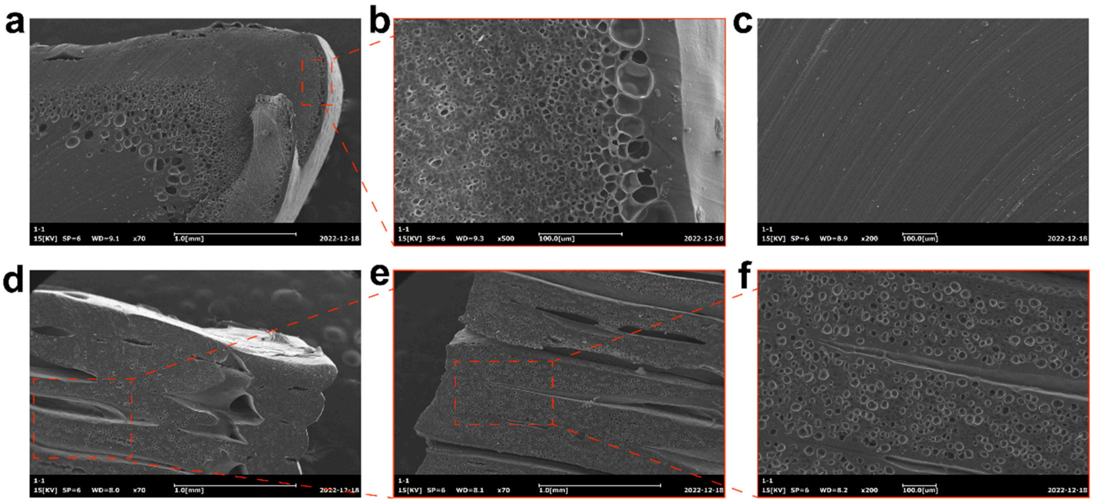

Figure S2. (a-c) SEM after foaming of pure TPU injection sample; and (d-f) SEM after foaming of printed sample with 90% filling density.

S3. Thermogravimetric analysis

260-330℃, the cracking of TPU; 360-500℃, collapse of the soft segment of the TPU; at 800℃, the carbon residue rate increased with the increase of MWCNTs content (Figure S3a).

When the weight loss is 50%, the decomposition temperature of the TPU blended with carbon nanotubes is higher than that of the pure TPU, which is due to the thermal barrier effect of the MWCNTs network (Figure S3b).

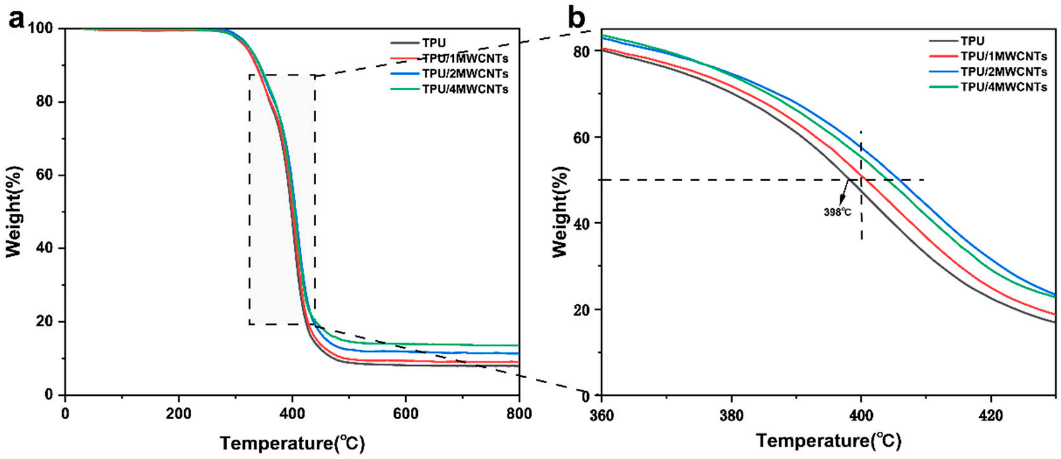

Figure S3. TG of TPU composites with different MWCNTs content.

S4. Printed and foamed samples

Figure S4a is the compression test sample model, honeycomb model of the lateral linear and curved interlayer bonding is good. Figure S4b is the tensile test sample, lateral and longitudinal interlayer bonding is good. Figure S4c is the pure TPU printed sample before and after foaming.

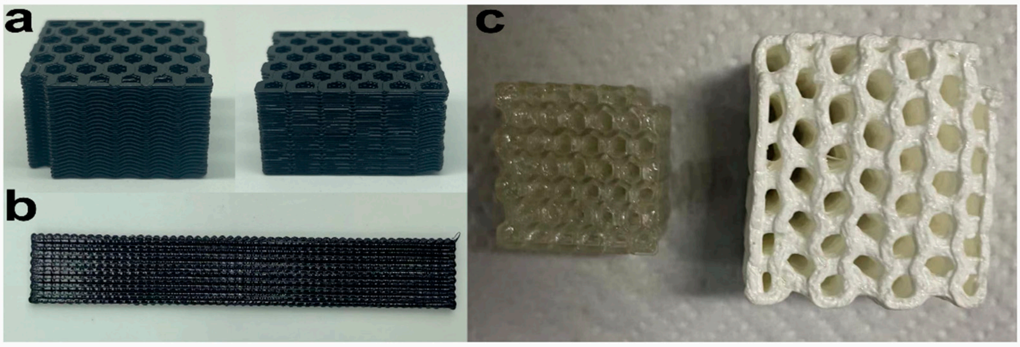

Figure S4. Printed and foamed samples.
